# Spinal Diseases

**Published:** 1824-09-01

**Authors:** 


### SPINAL DISEASES.

1. *Engravings Illustrative of a Work on the Nature and Treatment of the Distortions to which the Spine and the Bones of the Chest are Subject.* By JOHN SHAW, Surgeon, and Lecturer on Anatomy. Folio, pp. 47. Numerous plates. London, May, 1824.

2. *An Enquiry into the Causes of the Curvature of the Spine, with Suggestions as to the best means of preventing, or when formed, of removing the Lateral Curvature.* By T. JARROLD, M.D. 8vo. pp. 147. Two plates. London and Manchester, 1824.

IT is pretty evident, from the account of works on the spine (foreign and domestic) which we have laid before our readers, that the subject continues to engross a great deal of professional attention. The injurious effects of what we may term *forced cultivation* of the mind are now become so conspicuous that he who runs may read. But we apprehend that there are two great classes of causes in operation to produce spinal diseases—embellishment of the mind among the upper, and debasement of the body among the lower orders of society. By debasement of the body we mean the effects of sedentary and unhealthy occupations. The extent to which this last cause operates in manufacturing towns, and in all large cities, is beyond calculation or conception. The wide-spreading evil among the upper classes will shortly arrest the attention of every observer, and we have no doubt that the evil itself will be ultimately corrected by leading to gymnastic exercises by which the symmetry of the body may be preserved or restored. The ancients paid much attention to the means of effecting this desirable end, and we apprehend that the period is not far distant when the moderns will follow their example.

The letter press of Mr. Shaw's volume is so blended with, and illustrative of the plates, that it is quite impossible for us to offer any specimens here; but we cannot too strongly recommend the work to all surgeons, as containing a most rational and philosophic view of the subject, and pointing out clear and scientific methods of treatment.

In Dr. Jarrold's little volume will also be found much interesting matter, especially as relating to lateral curvature. When this last has existed for any length of time, it is so commonly accompanied by a feeble and emaciated frame as to warrant the conclusion that some specific disease exists—either as the cause or the consequence of the curvature. What is the nature of this disease, Dr. J. asks ?—

"Unless this is ascertained, and we know the source, we shall at best but palliate, not remove, the evil. Plans of cure have been proposed, without a reference to the cause of the affection they profess to remedy. The eye is struck with the fact that the Spine is bent, and upon this fact, solitary and uncombined, plans of cure have originated. One proposes a well contrived machine, to bear off the weight of the head, from the part which has protruded. Another proposes to accomplish the same end, by confinement to an horizontal posture for several successive months. A third recommends the carrying a weight upon the head, and by the exertion thus occasioned, to compel the muscles to force back again the yielding parts to their natural position." xi.

All these plans, he observes, relate only to the spine, "as having been mechanically curved, and can have no relation to the cause of the affection." We cannot quite agree with our author on this point. Those who attribute curvature of the spine to debility of the muscles have surely the cause of the affection in view, when they endeavour to restore the muscles to their proper tone. His observations apply indeed to mere mechanical contrivances. These can do little more than prevent the increase of the curvature till anchylosis of the vertebræ has taken place, when, in fact, the disease has run its course. Our author combats the doctrine advanced by Pott, that *scrofula* is the cause of spinal curvatures—and that of Mr. Wilson, that the curvature depends on *ricketts* or *mollities ossium*. He concludes that it is to a specific disease, and not to any modification of other diseases, that spinal curvature is owing—that it admits of the salutary operation of medicine—that it (lateral curvature) may be entirely prevented—or removed, if of recent occurrence.

Between lateral curvature, and curvature from within outwards there is much difference. The latter often begins in childhood or youth—sometimes in infancy or manhood. It is sometimes the consequence of accident, but more frequently of constitutional origin. It is generally attended with more pain, though less of indisposition, than lateral curvature. The vertebræ having reached their utmost point of projection and being anchylosed, form one mass, when the disease terminates. The strength and spirits which have been for some time improving, become sufficient for the business of life, and the constitution accommodating itself to existing circumstances admits the attainment of old age.

"When the body is inspected after death, the ligaments and cartilages are, in some cases, the only seat of complaint: these being destroyed, the heads of the superior vertebræ, fall obliquely on the vertebræ below; causing a slight elevation of the spinous processes. This early and natural termination of the disease, may frequently occur, and give credit to any plan of cure that may have been adopted. But it too generally happens, that, as well as the ligaments and cartilages, the bones also are in a state of caries; diminished and ragged like broken teeth." 23.

To machines and the horizontal posture, as remedial of this state, our author decidedly objects, but we cannot follow him through his arguments in opposition to these measures. We must refer to the work itself.

"The period required by the disease to pass through its stages, is as variable, as the curvature is to a greater or less degree extensive. Every year the curve is contracted at its base, and acquires a more conical form, until the disease terminates its course, which in many instances is completed in five years. The increase of the conical form, is determined by the rate, at which the caries diminishes the heads of the vertebræ, and gives them a wedge-like shape. While this process is going on, the ossification is necessarily incomplete, and the danger to life would be imminent, were not the loss of substance in the Spine, compensated by the strength acquired by its becoming a curvature." 28.

Our author, after relating some cases treated by occasional leeching and the internal use of hyoscyamus, soda, and alteratives, proceeds to "curvature of the spine with loss of motion of the lower extremities." To Mr. Pott we are indebted for the first ray of light on this subject. He shewed that paralysis and the loss of motion from a diseased spine were distinct and unrelated maladies—but this is all we know. An accidental ulcer that formed on the back of a youth confined with this complaint, led to the use of caustic issues. On this plan of treatment our author makes some critical remarks. Mr. Pott, he observes, does not shew on what principle caustic issues act. When they are applied to diseases of the joints, a specific object is in view—the reduction of inflammation and the prevention of suppuration. "But the latter, in caries of the vertebræ, seldom takes place; and did inflammation exist, it would be detected by the various dissections that have taken place. But I am not aware that any such appearances have been recorded." We grant that it is not ordinary or common inflammation that produces caries—but still we cannot conceive how caries can go on without being accompanied by *some kind of inflammation*. Our author grants that "there may be a peculiarity in the malady which issues will relieve—but not as in cases of ordinary inflammation." Dr. J. observes that Pott's plan of treatment has not maintained its reputation. "Their application (issues) has been general in all diseases of the spine, but their benefit extremely limited." Camper and Baynton considered issues as of doubtful efficacy—and this is probably the general opinion.

"Another mode of cure was recommended by Mr. Baynton, sanctioned by many instances of successful treatment. I mean that, of the Patient lying down on a couch for a length of time. If the view I have taken of the cause of the loss of motion of the lower extremities, being from the more than ordinarily rapid destruction of the intervertebral substance, and the consequent sudden declension of the vertebræ, occasioning a compression of the Spinal marrow, be correct, there is much propriety and excellence in the plan proposed by Mr. Baynton. Lying on a couch takes off the weight of the body from the diseased part, and retards the declension of the vertebræ; or when a curvature has commenced, and the lower limbs have lost their power, the composure and relief of a couch, enables the system to adapt itself to existing circumstances. And though no change may be made in the form of the Spine, yet such is the effort of the system to overcome obstacles, that, as in cases where a considerable blood vessel is destroyed, the part is kept alive by the smaller ones, and in a little time recovers its full sensibility and power: the same may be said of the nerves, if compressed. At first the effect is considerable, afterwards, life and power are gradually recovered. A white washer, learning his business, often complains of numbness of his hands, which being long held above his head, compress the nerves as they pass to the arm: but after some time, the numbness leaves him, and he pursues his avocation with the same ease, as if the arms were not raised above their natural elevation; so easily does the system overcome obstacles gradually introduced." 49.

Our author passes in review the Bath waters—the screw chair—the phosphate of lime, &c. concluding that caustic issues, the horizontal posture—and perhaps some well regulated machines are the only remedies that have at all maintained their ground. These, of course, are only calculated to counteract the consequences, not the cause of the disease. He thinks this last is deserving of attention. Although the opportunities our author has had of gaining a correct knowledge of the diagnostic symptoms of the approach of caries of the vertebræ, and of the means of opposing them have been too limited to pronounce them fully established, yet the public, he observes, are entitled to any remark that may lead to the relief of the afflicted.

"If, with a sense of weariness, there be a disposition to move the legs, and place them in various positions, accompanied by an uneasy sensation in the stomach and bowels, and a shortness of breath on ascending a hill, the state of the Spine should excite attention; but, if to these symptoms be added, a disposition to lie on the face, a countenance pale and gloomy, and a somewhat stiff and measured gait, an unequivocal evidence of the Spine being carious, will soon become apparent.

"In the early stage of the disease, the same means that are employed to restore a ricketed child to health, should be used, and all the vigour possible imparted to the system, by a diet consisting much of animal food, well seasoned with salt; at the same time correcting by medicine, any irregularities in the discharge of the functions of the body. Should the early indications of the disease escape due attention, or be misunderstood, and the commencement of pain in the back, show that caries is somewhat advanced; direct attention ought to be paid to the circumstance. If the vertebræ be not painful when pressed, the application of heat will be very grateful; if pain be excited by pressure, a few Leeches repeated as often as the pain increases, afford considerable relief. I have seldom thought it necessary to apply more than four at a time. Measures thus feeble, can only be beneficial on the supposition that the pain is chiefly occasioned by the swelling of the soft parts that surround the bone; and this is further supported by the patient not suffering acute pain when the back is bent, which must be the case, if the bone was in a state of acute inflammation. With a view of staying the progress of the disease, I have given Ext. Hyoscyami, with sufficient advantage, to induce me to recommend its use.

"When the vertebræ of the neck are the seat of the disease, the affection is manifested before caries has commenced, by the head being inclined to one side; in such cases, the Ext. Hyoscyami has in every instance in which it has been administered, proved beneficial, and in most effected a cure." 59.

Passing over the hip-joint disease, we come to lateral curvature of the spine—a disease certainly differing *toto cœlo* from that which has just been noticed. This is never the effect of accident—is not uniform in its progress—and never commences in infancy. The vertebræ do not become carious—purulent matter is not formed—the ligaments are not thickened—and death does not ensue, as an immediate consequence.

"Another alleged cause is, muscular debility; this opinion is advocated in the recent publication on the subject, and is the theory of the day. The effects of muscular debility cannot escape notice, they are seen in the infirm, in the aged, and the weak; but in them it does not produce a Curvature of the Spine. The body may be bent, the shoulders become round, a staff may be required, but the attitude thus occasioned is not that of an incurvated Spine. If debility be the cause, why is it local, why are the muscles attached to the Spine alone affected? are they paralized, or is the debility of a specific nature? if so, is it a disease, or by what other means is a local debility produced? The Spine is not drawn from its centre by relaxed and weakened muscles, it is constituted sufficiently strong to sustain the weight and motion of the head, and the action of the muscles, and is not in itself disposed to a change of form. If it be not one office of the muscles to keep the

Spine in its position; which without their effort would diverge, it is difficult to comprehend in what other way a weakness of the muscles can occasion a Curvature of the Spine." 78.

Our author's own opinion is, that lateral curvature depends on "a diseased state of the intervertebral cartilages." This diseased state is, he thinks, a thickening of their substance. "I have frequently seen, (says Mr. Wilson) the intervertebral substance thickened, and with such marks of vascularity, that I cannot doubt of ulceration occasionally taking place in them."

"Mr. Peter Barrow has favoured me with the following case.

'Jan. 9th, 1819, I visited Eliza Illingworth, aged nine years, who died a few hours after of inflammation of the chest. For more than eighteen months, this little girl, had been the subject of a curvature of the Spine, which extended to all the Dorsal vertebræ; the curvature was on the left side, and occasioned great deformity. No treatment had been had recourse to.

'On examination after death, the ligaments of the Dorsal part of the Spine, were slightly affected, but the intervertebral substances were greatly diseased, at some parts, particularly at the convexity, being red, and thickened; and at other parts, being converted into a pale jelly-like substance throughout, they had lost their elastic cartilaginous nature.'" 84.

In the view then which Dr. Jarrold takes of spinal curvature, a specific disease distends the intervertebral cartilages, which the energies of the constitution frequently remove, and the figure suffers only in degree;—in other and more advanced cases, the distension is so considerable as to occasion an uneasy sensation near the part, the effort to relieve which, causes the curvature—the muscles being made to act partially on the spine. On examination after death, we see no disease of bone, cartilage, or ligament. The cartilages and vertebræ are indeed compressed, so as to acquire the form of a wedge—"but this is the consequence, not a cause, the figure thus acquired, being the effect of pressure, not of disease." Nothing therefore that indicates the cause of the curvature appears. "Some cause without (exterior to) the spine bends it—and that cause is muscular action excited by the state of the cartilages." Our author being convinced that disease, not debility, originates the lateral curvature, proceeds thus:—

"But other evidence of the existence of disease yet remains; a curvature is commonly preceded by indisposition, more or less considerable, but without a definite character. After months or years passed in this state, its nature becomes manifest, by the enlargement of one of the shoulders; after which the indisposition diminishes, it being a termination of the attack; and in a few weeks, the shoulder becomes less, and the health improves; but the original shape is not fully restored, and those who suffer from this one attack, although the Spine be not incurvated, have lost something of the elegance of their figure. But commonly another attack follows, in the ensuing Spring, or it may be at the distance of years, when the flesh again wastes, the countenance becomes pale, the shoulder once more rapidly increases, but the indisposition is unwillingly acknowledged, because it is with difficulty described. All this may be completed in a few weeks, or even days, for the enlargement is not gradual, but sudden, I have seen a decided increase in a single day. Commonly on the third attack the Spine loses its natural position, and becomes slightly curved.

"Between the attacks the efforts of the individual assist those of nature to recover the loss of figure by calling the muscles into exercise which hold up the head, and draw back the shoulders; but the recovery is incomplete, Mr. Ward observes, "That the approaches of the curvature are for a long time scarcely perceptible; but on the occurrence of any particular disturbance of the constitution, such as a febrile indisposition, the spine, in the course of one, two, or three months, is found to yield in a greater degree than it had previously done, during as many years.'*

"This is an accurate description of an attack of the spinal disease, and is applicable to every attack which may occur in the life of an individual. The febrile affection, generally amounting only to a sense of lassitude, is not the cause of the curvature, for fever does not affect the spine, but is consequent on the existing state of the cartilages. Ask an individual advanced in life, whose spine is incurvated, the history of the malady, and the information will be, that the health had never been robust, that repeatedly, without any known cause, there had been an evident loss of flesh, with a sickly countenance, but without much personal indisposition; at other times the indisposition had been more marked. And that at these periods the curvature always increased, and although never in full health, yet there were seasons when there was an increase of spirits, and strength, during which, some progress was made in overcoming the curvature, but again it increased. If the enquiry be extended to others, the result will be, that some had suffered from only one attack; others from two; others from several. Each of which, permanently added to the loss of shape.

"Perhaps the curvature is never uniformly progressive, but is at periods stationary, and then as rapidly advances; it is therefore not mechanical. Neither the weight of the head, nor a relaxation of the muscles, can give it origin; because, their influence being uniform, and constant, so must also be their effects, and it is only by admitting the existence of a disease, that the Phenomena can be accounted for." 92.

The frequency of hereditary curvature of the spine, or disposition to it, is considered by our author as in favour of a specific disease.

* Ward's Practical Observations, p. 35.

"The first attack is commonly made before the age of fifteen, its approaches are so low, often insidious, and unobserved; exciting at its outset scarcely any feeling of indisposition, at least none are acknowledged, but such as are incident to growth, yet, the individual loses flesh, stoops, is dispirited, inactive, pale; the appetite is impaired, retirement from observation is sought, the deportment and habits are changed, the manners are become careless, ungraceful and spiritless, the temper is irritable, the apprehension dull, instruction is imparted with difficulty, and a spirit of self government, and laudable ambition so necessary in forming a character, attained in a very limited measure. If attention be paid to the form of the person, the cause is ascertained to be an incipient curvature of the spine.

"The shoulder blades are elevated, and prominent, so that the spine appears sunk between them, above them the flesh is considerably increased, and thickened, and is more than naturally firm and elastic. I beg particularly to call attention to this circumstance, as it is characteristic of the disease. In every one in whom I have noticed this appearance, although it may almost spontaneously disappear for a season, it has returned; and a curvature, unless counteracted by medicine, has always been the consequence. I repeat the remark, when the shoulder blades are somewhat elevated, and there is a greater quantity of flesh between them and the neck than corresponds with the flesh on the chest, a curvature if not prevented is inevitable. At this period, a short course of medicine replaces the shoulders in their proper position, and if future attacks, should be made, and be treated in a similar manner, the natural symmetry, and proportion of the figure, will be preserved. Remove the cause by destroying the disease, and nature will restore the figure." 97.

The first attack commonly runs its course unobserved. When over, the carriage of the head is more erect, and the shoulders recover much of their lost resemblance to each other. But still the scapulæ are elevated and project outward, so that the spine is sunk between them.

"A second attack is commonly attended by a sense of sinking at the stomach; a disposition to stoop which is with difficulty overcome; and by a pain under the shoulder-blade, to relieve which, the arm is frequently moved round in a circle, and the point of the shoulder thrust forward; and thus the shoulder-blade is drawn to a greater distance from the Spine, and becomes much larger than that on the other side; alarm is commonly now excited, and attention for the first time paid to the subject.

"This state of the shoulder-blade makes way for, and is the forerunner of the curvature. It will be said that this must be effected by muscular action, granted. By their strength the shoulder-blades are elevated, and drawn aside, and a continuation of the same influence ultimately incurvates the Spine. The controversy is not whether the muscles act, but how they are excited. To place the subject in another point of view; let the shoulders be voluntarily elevated, and projected forward, the trunk will then acquire much the same figure it has in the first attack of the Spinal disease. A little attention to the action of the muscles, will render it no longer problematical by what agency the curvature is effected, but unless the muscles had been excited, they would not have acted, a healthy Spine does not exert this influence, disease in some form is the exciting cause, and I have never been able to detect any but in the cartilages. In the second attack, the fulness at the upper part of the back again is evident; one of the hips also projects, and as has been remarked by some authors, is more than naturally covered with flesh. How far this bears a relation to the state of the shoulders, is not immediately apparent, as the projection of the hip may be supposed to be occasioned by the preponderance of the trunk to the right side.

"Every change in the form of the shoulders, effects a change in the centre of gravity, and consequently in the hips, and thus the air and gait in walking becomes peculiar and characteristic. The left foot is turned outward, and the knee scarcely bent, so that in walking, the limb is thrown round, rather than directed forward. The attention paid to the dress, much conceals the state of the shoulder; but a slight curvature is detected by the motion of the feet. The hip is never the seat of pain, but the shoulder in the second attack, is in no instance free, and is often pressed against the chair, or some other hard substance, to relieve from the weariness of a continued aching. The sitting down is always awry, and the left knee projects. In the subsequent attacks, less of fulness is observable in the shoulders, while the chest acquires a more natural state, but the health is more uniformly indisposed." 103.

We now come to the important question of prevention or cure. In bad cases—where the curvature is great and of some standing, it is useless to hope for a restoration of shape. But the health may be restored, and the figure somewhat improved. If by bending the body, the spine comes to its proper shape, we may promise a cure. Here our author reviews the various plans proposed by authors and practitioners—and wisely, we think, avoids rejecting any one of them as totally and always useless. From each, some auxiliary assistance may be gained—even from machines, when judiciously applied, some feeling of strength and comfort may be derived by the patient. Darwin advised the horizontal position, during a few hours each day—and this was extended by Baynton into a long and almost uninterrupted prostration for months in succession, in every stage and form of the disease. Success has, in some measure, justified the expectations of these distinguished advocates; but it must be confessed that disappointment has also been no uncommon occurrence.

"When the nature of the disease shall be better understood, the horizontal position will recover something of its reputation; for, as the disease does not from its own nature occasion a curvature, as the gout occasions distortion, the horizontal position must be advantageous as a preventive, or as opposing a further increase, or after the disease has been subdued; but, this position is seldom recommended till a curvature has been formed, and then, it is difficult to comprehend on what principle a cure can be effected. Every agent that can influence the form of the person is at rest, and a spontaneous cure is not probable." 111.

Of Mr. Wilson's plan (carrying a weight on the head) Dr. Jarrold speaks rather favourably. "When the disease has been subdued, this method may be adopted with considerable advantage, especially if combined with that recommended by Darwin; for the muscles, after being strongly excited in supporting the weight on the head, require absolute rest, that the advantage which had been gained, may not be lost in the relaxation incident on fatigue." Dr. J. has very often used Mr. Wilson's method with advantage. Shampooing, percussion, or manipulation, has its use in strengthening the muscles. We now come to our author's mode of treatment—or rather his addition to the modes of treatment already described, for he calls them all into his assistance occasionally.

"It has been my object to prove, that the lateral curvature has its origin in a specific constitutional disease, and consequently requires a constitutional remedy; and, as I before hinted, that there might be some relation between it and bronchocele, I have made use of similar remedies. From ten to fifteen grains of burnt sponge, and from four to six grains of carbonate of soda, and if debility be considerable, twenty drops of nitric acid, are directed to be given daily. Very soon, the increased flesh on the shoulders begins to diminish, and in two or three weeks, disappears. The shoulder blades at the same time fall, and reoccupy their natural situations; the health, which had been more or less disturbed, resumes its ordinary state; the mind becomes cheerful and capable of application; the languid, dispirited aspect, which seemed to call for the use of tonics, and stimulants, is dispelled without them. Medical treatment is seldom further required, unless the appetite, and digestion be impaired.

"When the first attack is allowed to run its course undisturbed, the consequences are by no means unimportant, the shoulder blades remain high and prominent, and no individual with high shoulder blades, can bear fatigue, or walk with ease. The centre of gravity acquires a new position, and the consequences before hinted at follow. I will repeat them: the form, and the carriage, is neither graceful nor dignified, the agility of the limbs is lessened. Fatigue is soon induced, and the constitution is reported to be weak and feeble; but this latter is an error. The natural constitution is, generally, very good; but the influence of a change in the centre of gravity, on the activity and strength, and the existence of a spinal disease, give the semblance of a bad constitution.

If, after the shoulders have acquired their natural appearance, the carriage is still awkward, let the child be induced to run, and the action of the feet will determine, whether the centre of gravity be in a proper, or a forced position. If the action wants freedom, the person will want strength and symmetry. In such a case, the supporting a weight on the head is highly beneficial; the evil must be overcome. In the second attack, the same means are used as in the first, but not with the same immediate and visible effect. The increased flesh, indeed, disappears, but the right shoulder is more prominent than the left, and the chest is narrow; the system is weak; the limbs are thin; medical aid is requisite. Individuals of a plethoric habit, are benefited by the loss of blood; others, whose digestive organs are impaired, require the ordinary treatment for that malady. When the spinal disease is removed, which is known by the diminution of the flesh on the shoulders, the attention is powerfully called to the figure, which has lost much of what contributed to its beauty and strength. This loss, the efforts of nature are, in general, incompetent to restore. But the assistance afforded the muscles, by the methods already recommended, is of essential importance; and, should the spine have been recently incurvated, and the power of recovering itself when the body is bent, not lost, persisting in these measures may complete a cure.

"In the next step of the disease, when the convex side of each vertebra is lessened from pressure, and that which was almost square has assumed the shape of a wedge, the recovery of the figure is impossible. But, even in this case, the disease may be subdued, the health may be recovered, the further progress of the curvature may be prevented. The benefit indeed is great, but the curve will remain; and the evil, though lessened, will still be an evil." 120.

Our author concludes his work with a short chapter on "anomalous affections of the spine"—a subject that opens a vast field for enquiry and observation, since many diseases that affect the spine, cannot be classed with either the lateral or outward curvatures. Our author's experience is not sufficient to do justice to the subject, yet he cannot pass it over entirely unnoticed.

The first which he notices, is that which affects the muscles of the neck, so as to destroy the influence of the will upon them. The term paralytic is not applicable to the malady. The head rests on one shoulder, or inclines in some other way, altogether uncontrollable by the will. No contiguous muscles partake of the weakness—"nor have those of the neck the deadness of common palsy."

"The affection sometimes commences with a sense of suffocation, or breathlessness, except while in the open air. But in other cases, the loss of muscular power is not preceded by any symptom of bad health; what first gives rise to suspicion is, the head being involuntarily drawn aside, when the attention is excited, as in reading; but which is recovered, on the attention being withdrawn. But the indisposition still remains; and very soon reading becomes unpleasant, from the contraction or weakness of the muscles, and is given up. Still the disease advances; the head inclining more and more, till no power remains in the will to direct the muscles.

"In some cases all the muscles of the neck are affected, in others, only those of one side, which occasions a painful rigidity of the muscles on the opposite side, they being excited to action, while their antagonist muscles are paralyzed; thus drawing the head with considerable force in the line of their motion, occasioning distress from the appearance it produces, and the pain it excites" 132.

This affection is not permanent, for it runs its course in a few years, or a much shorter period, and strength is restored. If any thing has been used towards the close of the complaint, it gains the credit of curing it. The disorder often returns, and again disappears. "It has been immediately followed by insanity—sometimes by death."

Another class of anomalous spinal affections is characterized by one or more years of indisposition—often attributed to debility, or to worms, or to some affection of the brain. The spine is very flexible, and the individual is indisposed to stand erect, so that, when examined, the spine is seldom straight. Change of air, and nutritious diet, are generally prescribed, but with little benefit.

"The sense of debility continues, till a pain of the back sends the individual in a state of great helplessness, to the bed from which, she is not soon to rise. The pain is commonly confined to one, or two vertebræ, though it is not stationary, but attacks the lower vertebræ, then the higher, or removes to the head.

"In some cases the whole of the spine is painful, but not uniformly so; for certain of the vertebræ are alternately more acutely sensible. Independent of the pain, there is a sense of debility, or rather an incapacity of bearing the erect posture without fainting. The countenance, in the midst of this suffering, is not dejected, or sickly." 135.

Some cases are related, in illustration of which we can only afford space for the particulars of one.

Miss M——, aged 15 years, complained, in 1820, of much fatigue after exercise—of impaired appetite, and depressed spirits. These symptoms continued more than a year, when an afflictive event removed her from school. Soon afterwards her back became painful, and in a few weeks confined her to bed. The third dorsal vertebra was exquisitely sensible to the touch. In a few days the pain removed to the second lumbar vertebra, which it never entirely left. But a more acute and occasional pain was perceived in some other part of the spine, or in the head. The countenance was not depressed, nor the mind weakened. The bowels were inactive, and the discharges black and offensive. Removal from the bed occasioned fainting. This state continued from the 14th March, 1821, till September of the same year, when she was able to sit in an easy chair for a few minutes. She has, since that time, gradually improved in strength, and is now able to walk some miles. Purgatives and repeated bleedings afforded some relief; but our author apprehends that Nature effected a cure. The parts of the spine that were affected, slightly protruded, and remained so for some time after the pain subsided, but the protrusion has since disappeared.

"This class is characterized by the extreme helplessness of those affected. The erect position can never be borne, except for a short period; while the countenance does not indicate the existence of disease, but has very much of its natural appearance. In most of the cases that I have been made acquainted with, some strong excitement of the mind has preceded the attack. In others, insanity has accompanied it; and, as the Spine never permanently loses its natural form, it may not be unworthy of enquiry, whether the brain, and Spinal marrow, be not the seat, both of this, and of the preceding class of diseases." 189.

A third class of these anomalous affections will be illustrated by the following short cases.

"Ann Barnes, aged 7, 1816, had been observed for several months to decline in strength. At first her step was short, and guarded; then she could only walk by taking hold of something, for support; at length her lower extremities became useless. I saw her, Feb. 7, when, in addition to an incapacity to move the legs, the whole back was bent, so as to form an arch; and she was wholly unable to hold herself upright. I prescribed two grains of Calomel, to be given every morning. On the 15th, she could move her limbs. 21. The back was less bowed, and the limbs stronger. 28. She could support herself, and by the end of March, her strength and figure were recovered.

"Master M. aged 4, 1820, after being able to walk, lost the power. I saw him Aug. 4. The abdomen was hard, and ful; the tongue furred. The breath offensive. The Spine formed the segment of a circle, without the child being able, except by lying down, to change that form; and even then, the back was not straight. I prescribed a grain of Calomel, followed by a tea spoonful of Magnesia, every morning.

"No perceptible benefit was obtained, till the expiration of the third week, but, after that period, the improvement was uniform, and in three months, the child was well. The medicine was occasionally varied, but the sole object was to correct the state of the bowels." 145.

This affection of the spine, our author considers as evidently sympathetic of the state of the stomach and bowels—and consequently is, in many cases, cured only by a long course of laxative medicines—which if steadily persevered in, have never failed in our author's experience.

"The last variety of Spinal affections that have occurred to me, in sufficient number to form a distinct order, is that change in the figure of the Spine, which is connected with a globular chest. This affection is occasioned by difficult respiration, which in childhood, is commonly attributed to inflammation, and means that debilitate are used as a cure; often with present success. But the disease returns in a few months, and is more obstinate. Again, it is temporally removed, and the child acquires the character of being highly inflammatory.

"The disease however, is in the digestive organs, and is more effectually subdued, by increasing their strength. After repeated fits of laborious breathing, the chest acquires a globular form, from the action of the muscles, to which the Spine corresponds." 147.

We have now presented our readers with a full account of the little work before us. It is evidently the result of considerable observation and reflection; and although it is defective in the scientific anatomical and physiological details by which Mr. Shaw's work is characterized, yet it is very useful in its way. On such an abstruse and little cultivated subject we want labourers of various and different kinds. We want close and attentive observers of external phenomena—good anatomists—ingenious mechanists—and even dexterous shampooers. None of these are to be relied on exclusively—none of them are to be rejected contemptuously by the prudent eclectic practitioner.

